# An integrative method for scoring candidate genes from association studies: application to warfarin dosing

**DOI:** 10.1186/1471-2105-11-S9-S9

**Published:** 2010-10-28

**Authors:** Nicholas P Tatonetti, Joel T Dudley, Hersh Sagreiya, Atul J Butte, Russ B Altman

**Affiliations:** 1 Biomedical Informatics Training Program, Stanford University School of Medicine, Stanford, CA, USA; 2 Departments of Pediatrics and Cancer Biology, Stanford University School of Medicine, Stanford, CA, USA; 3 Stanford University School of Medicine, Stanford, CA, USA; 4 Departments of Bioengineering and Genetics, Stanford, CA 94305-5479, USA

## Abstract

**Background:**

A key challenge in pharmacogenomics is the identification of genes whose variants contribute to drug response phenotypes, which can include severe adverse effects. Pharmacogenomics GWAS attempt to elucidate genotypes predictive of drug response. However, the size of these studies has severely limited their power and potential application. We propose a novel knowledge integration and SNP aggregation approach for identifying genes impacting drug response. Our SNP aggregation method characterizes the degree to which uncommon alleles of a gene are associated with drug response. We first use pre-existing knowledge sources to rank pharmacogenes by their likelihood to affect drug response. We then define a summary score for each gene based on allele frequencies and train linear and logistic regression classifiers to predict drug response phenotypes.

**Results:**

We applied our method to a published warfarin GWAS data set comprising 181 individuals. We find that our method can increase the power of the GWAS to identify both VKORC1 and CYP2C9 as warfarin pharmacogenes, where the original analysis had only identified VKORC1. Additionally, we find that our method can be used to discriminate between low-dose (AUROC=0.886) and high-dose (AUROC=0.764) responders.

**Conclusions:**

Our method offers a new route for candidate pharmacogene discovery from pharmacogenomics GWAS, and serves as a foundation for future work in methods for predictive pharmacogenomics.

## Background

Genome-wide association studies (GWAS), which generally assay common variation and rely on relatively large effects, are now being widely applied to perform genome-wide scans for variants associated with pharmacological phenotypes [1,2]. In the case of complex diseases, GWAS have yielded associated variants with modest overall effects. However, in the case of pharmacological phenotypes, initial results from pharmacogenomic GWAS appear to indicate a greater ability to discover variants with substantial effect size [1,3]. Nevertheless, pharmacogenomic GWAS suffer many of the limitations of disease GWAS in that follow up studies are often required to elucidate the causative genes and variants latent in the GWAS results [4-6]. Additionally, pharmacogenomic GWAS are also limited in power by small cohort sizes [7]. Amongst the drugs whose pharmacological variance has been evaluated using the GWAS approach, warfarin (Coumadin) has emerged as a prominent pharmacogenomics case study with great translational potential.

Given the its broad use, narrow therapeutic range, and severity of side effects, a comprehensive pharmacogenomic characterization of warfarin dose-response offers the potential for substantial clinical impact [8,9]. Retrospective studies revealed the role of VKORC1, CYP2C9, and CYP4F2 in mediating abnormal variations in warfarin dose response [10-12], explaining approximately 30%, 10% and 5% of the variance in drug response respectively [13]. The relationship between variants in these genes and atypical warfarin dose response has been subsequently confirmed by GWAS analysis [14,15]. Recently, a method to estimate stable warfarin dose was developed by integrating information on patient VKORC1 and CYP2C9 genotypes with clinical factors [16]. Although this method incorporated genotype information for only two warfarin pharmacogenes, the genotype-based method was able to explain ~49% of the variance in stable dose, substantially outperforming the pure-clinical and fixed-dose approaches. The elucidation of additional large-effect pharmacogenes for warfarin and other genomic features might serve to dramatically increase the ability to predict warfarin dose response from genotype.

Therefore, in this study, we propose a novel method for increasing the power of pharmacogenomic GWAS and detecting pharmacogenes predictive of drug response. Our method characterizes the degree to which uncommon alleles of a gene correlate with drug response. If uncommon alleles are associated with atypical drug response phenotypes, then the gene is considered a predictive pharmacogene and a putative marker for drug response.

## Results

### Using knowledge to limit the hypothesis testing space

We identified 228 genes which were likely to be pharmacogenes for warfarin and which also contained SNPs measured in the Cooper warfarin response GWAS data set (See Methods). There were 3,856 SNPs contained within these 228 genes. We tested each of the 3,856 SNPs with a univariate linear regression model for its ability to predict warfarin dosage, the exact analysis performed by Cooper, et al., however, with the advantage of testing fewer hypotheses. As expected, the results of this analysis closely resemble the results of the Cooper analysis except that a much less strict significance threshold is necessary to correct for multiple hypothesis testing (1.3e-5 as opposed to 1.0e-7). However, even with this lower threshold only one SNP, rs10871454 (VKORC1), was significant after correction (Table 1).

**Table 1:** Knowledge filtered significant SNPs

SNP	Gene	Name	P-Value
**rs10871454**	**VKORC1**	**Vitamin K1 2,3-epoxide reductase subunit 1**	**9.31E-10**
rs4086116	CYP2C9	cytochrome P450, family 2, subfamily C	7.54E-05
rs4917639	CYP2C9	cytochrome P450, family 2, subfamily C	9.47E-05
rs9332169	CYP2C9	cytochrome P450, family 2, subfamily C	2.33E-04
rs9332214	CYP2C9	cytochrome P450, family 2, subfamily C	2.33E-04
rs10509680	CYP2C9	cytochrome P450, family 2, subfamily C	2.33E-04
rs12445568	HSD3B7	3-beta-HSD VII	3.84E-04
rs12357515	EXOC6	Exocyst complex component Sec15A	3.86E-04
rs11187215	EXOC6	Exocyst complex component Sec15A	3.87E-04
rs7294	VKORC1	Vitamin K1 2,3-epoxide reductase subunit 1	4.15E-04

Top 10 of 3,856 SNPs fitting a univariate linear regression model of warfarin dose for 181 patients. Bold SNPs are significant after multiple hypothesis testing. Only rs10871454 (a SNP in 100% LD with VKORC1) was significant after correcting for multiple hypothesis testing.

### Derivation and evaluation of a candidate gene-score based on allele frequencies

W assigned each SNP to a gene if the SNP was within 5 kbp of the boundary of the gene. Some SNPs mapped to more than one gene. We then aggregated the SNPs into genes using this mapping and calculate each gene’s pHap gene-scores (See Methods). Each gene-score was then tested with a univariate linear regression model for its ability to predict warfarin dosage, again, the same analysis performed by Cooper, except that we now are testing only 228 hypotheses and each gene-score is a summary of a set of SNPs frequencies. In this analysis both VKORC1 and CYP2C9 are significant for predicting stable warfarin dosage (Table 2). VKORC1 and CYP2C9 pass the corrected significance threshold of 1e-3 with p-values of 9.1e-7 and 9.6e-5 respectively.

**Table 2:** Univariate gene linear regression

Gene	Name	P-Value
**VKORC1**	**Vitamin K1 2,3-epoxide reductase subunit 1**	**9.08E-07**
**CYP2C9**	**cytochrome P450, family 2, subfamily C**	**9.63E-05**
NSUN6	NOL1/NOP2/Sun domain family 6	1.07E-02
UBE3A	E6AP ubiquitin-protein ligase	1.35E-02
BRF1	B - related factor 1	1.39E-02
QTRTD1	queuine tRNA-ribosyltransferase domain containing 1	1.54E-02
F8	Procoagulant component	2.40E-02
BAT5	HLA-B associated transcript 5	3.02E-02
COL1A2	Alpha-2 type I collagen	3.23E-02
RCN2	E6-binding protein	3.27E-02

Genes with candidate gene-scores that significantly predict dose in a univariate linear regression model (p≤0.05). Genes in bold are significant after correcting for multiple hypothesis testing.

### Extreme dose response warfarin response models

The gene-score was calculated for each of the 228 genes in the WSP for each patient in two classes: low dose patients and the compliment. The distributions of gene-scores of the two classes were tested for the null hypothesis, namely that the means of the distributions were equal. Two genes significantly distinguish the two classes after corrected for multiple hypothesis testing, VKORC1 and UBE3A with p-values of 1.2e-4 and 5.4e-5 respectively. 18 other genes had p-values that were less than 0.05, but not significant after multiple hypothesis testing (Table 3).

**Table 3:** Low/not low model

Gene	Name	P-Value
**UBE3A**	**E6AP ubiquitin-protein ligase**	**5.39E-05**
**VKORC1**	**Vitamin K1 2,3-epoxide reductase subunit 1**	**1.16E-04**
SLA2	Src-like adapter protein 2	2.11E-03
DICER1	Dicer1, Dcr-1 homolog	3.76E-03
CYP2C9	cytochrome P450, family 2, subfamily C	5.49E-03
SLC22A1	solute carrier family 22	1.12E-02
BBC3	BCL2 binding component 3	1.49E-02
F8	Procoagulant component	1.74E-02
HMOX2	heme oxygenase (decycling) 2	1.79E-02
MUTED	muted homolog	1.99E-02
VWF	coagulation factor VIII VWF	2.40E-02
HSD3B7	3-beta-HSD VII	2.56E-02
SPIN1	spindlin 1	2.58E-02
SELPLG	selectin P ligand	2.89E-02
FAM113B	family with sequence similarity 113, member B	2.92E-02
F13B	Fibrin-stabilizing factor B subunit	3.36E-02
MVP	major vault protein	3.39E-02
UGT2B7	UDP glucuronosyltransferase 2B7	3.83E-02
AKR7A2	aflatoxin beta1 aldehyde reductase	4.17E-02
SERTAD1	CDK4-binding protein p34SEI	4.96E-02

Top 20 genes with candidate gene-scores that significantly distinguish between low dose patients and non-low-dose patients (p≤0.05). P-Values are result of t test between low-dose and non-low-dose distributions of gene-scores. Genes in bold are significant after correcting for multiple hypothesis testing.

**Figure 1:**
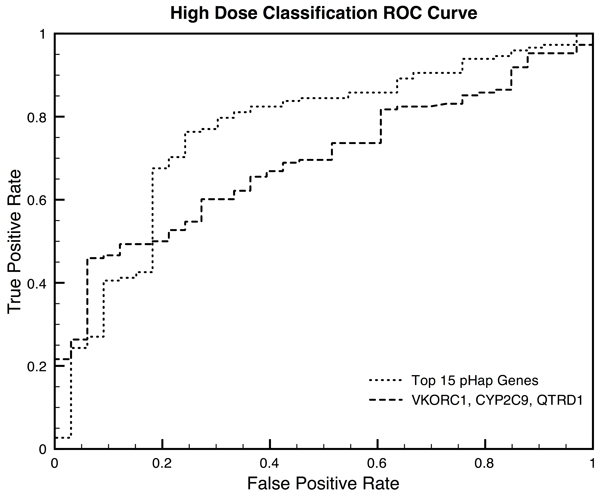
**Low Dose Classification ROC Curve.** Receiver Operating Characteristic Curve for the low dose classification algorithms. Two classifiers were trained, the first, dotted line, on all 20 genes for which the gene-scores significantly distinguish low-dose and non-low-dose patients (AUROC=0.886, p≤0.05, Table 3), and the second, dashed line, on only those genes that were significant after multiple hypothesis testing correction (AUROC=0.721, p≤0.001, Table 3). Both classifiers have empirical p-value significance of less than 0.01 when tested using bootstrapping.

A logistic regression classification model was trained on VKORC1 and UBE3A gene-scores and evaluated with 10-fold cross validation (Figure 1). The AUROC was 0.721 (significance p-value < 0.01). A second logistic regression classification model was trained on the 20 genes that had a p-value ≤ 0.05 (Figure 1). The AUROC of this classifier was 0.886 (significance p-value < 0.01).

Analogously for the high-dose model, three genes were found to have significant p-values after multiple hypothesis testing, VKORC1, CYP2C9, and QTRTD1: p-values of 4.4e-6, 2.3e-4, and 3.2e-4 respectively. 12 other genes had p-values ≤ 0.05 before multiple hypothesis correction (Table 4).

**Table 4:** High/not high model

Gene	Name	P-Value
**VKORC1**	**Vitamin K1 2,3-epoxide reductase subunit 1**	**4.39E-06**
**CYP2C9**	**cytochrome P450, family 2, subfamily C**	**2.27E-04**
**QTRTD1**	**queuine tRNA-ribosyltransferase domain**	**3.21E-04**
NAT13	N-acetyltransferase 13	5.25E-03
BAT5	HLA-B associated transcript 5	6.76E-03
CRP	C-reactive protein, pentraxin-related	1.02E-02
COL1A2	Alpha-2 type I collagen	1.37E-02
GGCX	Vitamin K gamma glutamyl carboxylase	1.44E-02
A2M	C3 and PZP-like alpha-2-macroglobulin	2.06E-02
FBXO28	F-box protein 28	2.14E-02
HSD3B7	3-beta-HSD VII	2.54E-02
ITGA5	Fibronectin receptor subunit alpha	4.07E-02
ALS2	amyotrophic lateral sclerosis 2	4.13E-02
NSUN6	NOL1/NOP2/Sun domain family 6	4.21E-02
SORBS3	sorbin and SH3 domain containing 3	4.99E-02

Top 15 genes with candidate gene-scores that significantly distinguish between high dose patients and non-high-dose patients (p≤0.05). P-Values are result of t test between low-dose and non-low-dose distributions of gene-scores. Genes in bold are significant after correcting for multiple hypothesis testing.

**Figure 2:**
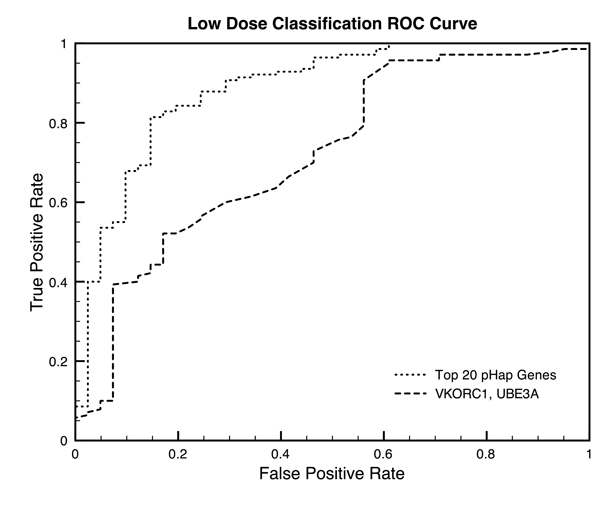
**High Dose Classification ROC Curve**. Receiver Operating Characteristic Curve for the high dose classification algorithms. Two classifiers were trained, the first, dotted line, on all 15 genes for which the gene-scores significantly distinguish high-dose and non-high-dose patients (AUROC=0.764, p≤0.05, Table 3), and the second, dashed line, on only those genes that were significant after multiple hypothesis testing correction (AUROC=0.693, p≤0.001, Table 3). Both classifiers have empirical p-value significance of less than 0.01 when tested using bootstrapping.

A logistic regression classification model was trained on VKORC1, CYP2C9, and QTRTD1 and evaluated with 10-fold cross validation (Figure 2). The AUROC was 0.693 (significance p-value < 0.01). A second logistic regression classification model was trained on the 15 genes that had a p-value ≤0.05 (Figure 2). The AUROC of this classifier was 0.764 (significance p-value < 0.01).

## Discussion

In this study we present a novel method for integrating pre-existing pharmacological knowledge and scoring candidate genes from association studies. We have demonstrated that this filtering and scoring method is capable of identifying candidate genes explaining a large proportion of warfarin dose response phenotypes.

Our pHap value is a simple measure of how “extreme” an individual’s variants are in a particular gene: if all the SNPs show minor alleles, then the individual has a very high pHap value for that gene. If all the SNPs show major alleles, then the individual has a low value for that gene. We have shown that this aggregate measure of genotype has the advantage of aggregating genetic variation in order to reduce the number of hypotheses tested. This measure cannot only increase the power to identify candidate genes that explain dosage variation but also can identify candidate genes for extreme phenotypes.

We validate our methods on the warfarin GWAS study by Cooper et al, which attempted to identify candidate genes by examining a SNPs ability to predict dosage in a univariate linear regression model. The power of the Cooper analysis, however, was limited by the low number of patients in the cohort, a common problem in drug GWAS[7]. When our methods were applied to the same data set we were able to identify the two best characterized genes responsible for variation in warfarin dose response, VKORC1 and CYP2C9. It is interesting to note that the knowledge filtration method alone will not identify both of these two genes (Table 1). The pHap, which summarizes the aggregate contribution of a set of SNPs, is also needed in order to identify CYP2C9 significantly (Table 2).

We also show that our method can significantly identify features for a machine learning classification algorithm. We achieve high performance when classifying between low/not low-dose patients and between high/not-high dose patients (AUROCs of 0.886 and 0.764 respectively, Figures 1 and 2). Using an empirical bootstrapping approach we demonstrated that the genes our method identifies are significant. In both cases the AUROC of the model was the highest observed, corresponding to a p-value < 0.01.

We acknowledge that the described method is dependant being able to identify potentially important pharmacogenes. It is important to note that the algorithm we employed to rank genes for their potential to be pharmacogenomic does not require the drug to be previously known and will predict pharmacogenes for novel chemical structures.

The results of this study offer support for the applicability of allele-based pharmacogenetic models for the prediction of drug response phenotype. In addition, it opens up new avenues for candidate pharmacogene discovery. In future work we plan to improve our method through the investigation of more sophisticated classification methods and identification of additional genetic and genomic features predictive of drug response. We will also seek to expand the application of our method to additional drugs, with the overarching aim of developing a method that is predictive and robust across a broad range of drugs. We also plan to investigate the warfarin-associated pharmacogenes identified by our approach and work to biologically validate their putative role in warfarin response.

## Conclusions

We have developed a novel approach that incorporates pharmacogenomic knowledge integration and a gene scoring system based on SNP aggregation to enhance pharmacogene discovery from pharmacogenomics GWAS. We applied this approach to a published warfarin GWAS data set comprising 181 individuals and found that our method can increase the power of the GWAS to identify established warfarin pharmacogenes VKORC1 and CYP2C9, and implicate several novel warfarin pharmacogenes. Additionally, we find that our method can be used to discriminate between low-dose (AUROC=0.886) and high-dose (AUROC=0.764) responders, establishing a basis of direct clinical utility for the approach. Based on the performance observed with the warfarin GWAS, we recommend future work to extend the approach and apply it to additional pharmacogenomic GWAS as well as GWAS characterising other traits of clinical interest.

## Methods

### Warfarin GWAS data

For validation, a dataset of 181 patient genotypes (175 Caucasian, 6 Hispanic) and stable warfarin dosages was obtained from a warfarin GWAS study described by Cooper et al.[15]. The list of SNPs measured by the Cooper et al. study was filtered so that the set of SNPs had a maximum pairwise r-squared linkage disequilibrium score of 0.2. The resulting set was then queried against the SCAN SNP and CNV Annotation Database to determine whether the SNPs were either contained within a given gene or within 5kbp upstream or downstream of that gene. One exception to the SCAN SNP mapping was made so that rs10871454 mapped to VKORC1. This SNP is in perfect linkage disequilibrium with rs9923231, a VKORC1 SNP[15].

### Using knowledge to limit the hypothesis testing space

GWAS studies are hindered by multiple hypothesis testing corrections that significantly limit the power of analysis on smaller patient data sets. Pharmacogenomic knowledge bases integrate large amounts of data from the literature and biological experiments. Recently, an algorithm, called the PGxPipeline, was published that integrates pharmacogenomics and drug-binding databases to rank genes for their likelihood to be pharmacogenomic for a given drug[17]. The PGxPipeline was used to rank genes for their likelihood to be involved in the pharmacokinetics or pharmacodynamics of warfarin metabolism and action. This ranking of genes was then repeated for each of 486 other drugs for which pharmacogenetic interactions are known. These other drug gene rankings were then used to apply a significance score to the warfarin gene rankings. Only the most significant (p-value ≤ 0.05) warfarin genes were used the following analysis. 786 such genes were identified to have significant likelihood scores to be potential warfarin pharmacogenes. Of those, 228 contained SNPs that were measured by the Cooper GWAS study. We define this genes set to be the Warfarin-Specific Pharmacogenome (WSP). We limited our analysis to just those 3,856 SNPs that were in these 228 genes. The univariate linear regression analysis used in the Cooper study was repeated for these 3,856 SNPs (Table 1).

### Derivation and evaluation of a candidate gene-score based on allele frequencies

We aggregated he 3,856 SNPs by gene and a computed the gene-score for each gene based on the SNP allele frequencies. These features are based on the straightforward assumption that genes with a preponderance of low-frequency alleles in individuals with extreme drug response phenotypes are more likely to be modulating that response[18]. Variants of pharmacogenes are important in determining drug response. As a proxy to the potential of variants in a pharmacogene to effect drug response we define the pHap score. To calculate the pHap score for a given patient and a given gene we first compute the negative log of each genotype frequency for each SNP in the given gene. We then take the sum of these values and call this the pHap score. The pHap score for patient, *i*, and gene, *j*, is defined as

where *N_j_* is the number of SNPs in gene *j* and *f_i,j,k_* is the frequency of the allele of patient *i* for SNP *k* of gene *j*. Therefore, there is a pHap score for each gene in the WSP for each patient. In total we generated 228 pHap scores for each patient (for the 228 included genes). We then fit each gene-score in a univariate linear regression model to the dosage data and tested for the gene-score’s ability to predict the dosage data (Table 2).

### Redefinition to a classification problem

To test the method’s ability to identify genomic features that are predictive of phenotypes the patients were divided into classes based on their stable dose. The first two classes consisted of patients who required a low stable dose of warfarin (≤3mg/day) and the compliment set of patients. The latter two classes consisted of patients who required a high stable dose of warfarin (≥7mg/day) and the compliment set of patients. Dividing the patients into these two sets of classes (low/not low and high/not high) redefines the task as a classification problem. This allows us to train machine-learning algorithms on the features identified by our method and, if the features are discriminatory, predict the drug response classification of the patient.

### Identification of features to distinguish extreme dose patients

Patients were divided into two classes, those that required a low stable dose of warfarin and those who did not. For each patient we calculated the gene-score of each gene and tested for the two classes of scores for the null hypothesis using a Students *t* test. Since 228 null hypotheses were being tested the p-values were corrected using the conservative Bonferroni multiple hypothesis correction method. The new threshold for significance was set at 0.001 (Table 3).

We trained two logistic regression classifiers. The first classifier we trained was on only the genes that were significant after correcting for multiple hypothesis testing and the second was trained on all genes that had a p-value ≤ 0.05. The classifiers were evaluated using 10-fold cross validation. In 10-fold cross validation the classifier is trained on 9/10th of the data and the remaining 1/10th of the data is reserved to evaluate the performance of the classifier. This process is repeated 10 times and the average performance is plotted in a receiver operating characteristic (ROC) curve. The area under the ROC curve (AUROC) is a summary statistic for overall the performance of the classifier (Figure 1).

In order to evaluate the significance of the classification models an empirical p-value was derived. To derive an empirical p-value, we trained the classifiers on randomly chosen genes from the set of 228 in the WSP. The number of genes chosen at random corresponded to the number of genes used in the two classifiers. We repeated this process 100 times for each classifier.

The analogous analysis was repeated for the high-dose/not-high-dose patient classifications (Table 4, Figure 2).

## Authors' contributions

NPT and JTD designed the study, carried out the analysis and wrote the paper. HS contributed the data and provided critical feedback on the paper. AJB contributed critical analysis of study design and the paper. RBA participated in the study design and wrote the paper.

## Competing interests

The authors declare that they have no competing interests.
